# Relationship between psychological adaptability and work engagement of college teachers within smart teaching environments: the mediating role of digital information literacy self-efficacy

**DOI:** 10.3389/fpsyg.2023.1057158

**Published:** 2023-06-27

**Authors:** Shiyi Fan, Zeyuan Yu, Xin Zheng, Chunhai Gao

**Affiliations:** ^1^Faculty of Education, Southwest University, Chongqing, China; ^2^Department of Faculty Affairs, Chongqing Jiaotong University, Chongqing, China; ^3^Faculty of Education, Shenzhen University, Shenzhen, Guangdong, China

**Keywords:** smart teaching, psychological adaptability, work engagement, digital information literacy, self-efficacy

## Abstract

**Introduction:**

Integrating the Internet and traditional teaching has enriched teaching resources and methods and introduced many advanced digital media. The smart teaching process is influenced by teachers' psychological adaptability, which can be affected by teachers' work engagement. However, the relationship between the two has not received sufficient attention in the literature. This study aims to analyze the relationship between college teachers' psychological adaptability and work engagement in a smart teaching environment.

**Methods:**

Applying structural equation modeling (SEM) to a sample of 373 front-line teachers, this study focuses on the mediating effect of digital information literacy self-efficacy on the relationship between teachers' psychological adaptability and work engagement.

**Results:**

The results show that the four dimensions of college teachers' psychological adaptability strongly influence work engagement and digital information literacy self-efficacy. In particular, teachers' psychological adaptability and work engagement are positively correlated; teachers' self-efficacy can positively affect the three dimensions of their work engagement, and teachers' psychological adaptability can positively affect their digital information literacy self-efficacy.

**Conclusion:**

The above results can serve as a basis for the development and improvement of the training of college teachers and the implementation of smart teaching. The study findings highlight the importance of training teachers on information technology teaching and implementing measures to enhance teachers' digital information literacy self-efficacy. Training should focus on the knowledge and skills of teachers using information technology teaching and increase the practical links of teachers using information technology teaching.

## 1. Introduction

With the rapid development of the Internet, many technology-based network companies have entered the education industry. The integration of the Internet and traditional teaching has enriched teaching resources and methods and introduced many advanced digital media (Qiao, 2021). Smart teaching aims to enable teachers to apply efficient teaching methods by constructing a learning environment that integrates technology, cultivating talents with a proper value orientation, strong action capabilities, excellent thinking quality, and profound creative potential (Kalugina and Tarasevich, 2018). Four supporting points have been argued as necessary to promote smart teaching: an educational philosophy conducive to personality development, an open and diverse cultural form, a fair and complete education system, and high-quality educational resources (Kochkorbaevna and Hilola, 2022). Over the years, information technology has helped education and has yielded considerable effects, although its influence is still limited to a small portion of schools. “Smart technologies are technologies that are transferred to procedures based on interaction and exchange of experience” (Achilovich, 2021, p. 133–137). The in-depth integration of information technology and innovative design concepts can reflect the idea of “smart” throughout the entire teaching process, improving the teaching and learning interactions and experiences between teachers and students in the current teaching scenarios, and making the traditional single teaching model more diverse and flexible (Biwer et al., 2020).

Information technology awareness refers to the subjective willingness of teachers to use information technology in educational activities to improve educational achievements by integrating information technology into teaching goals (Rashidov, 2020). Teachers' self-efficacy refers to their judgments, beliefs, and feelings about the value of education, the educational capabilities of doing an excellent job themselves, and the positive influence on students' development (Perera et al., 2019). It encompasses the general view of teachers on the overall educational value, the status and role of education in students' development, and the overall relationship between teachers and students (Joo et al., 2018). Teachers' digital information literacy self-efficacy can influence their psychology and work engagement, as has been reported in many studies (Zou et al., 2021).

### 1.1. Research gaps

As a main body of teaching professionals, teachers play an influential role in smart teaching. The smart teaching process is influenced by teachers' psychological adaptability and has been shown to have a link with teachers' work engagement. However, the relationship between the two has not received sufficient attention in the literature, especially from an Asian context.

Also, some studies exploring smart teaching have analyzed the relationship between psychological adaptability and the work engagement of college teachers (Collie et al., 2020a,b; Holliman et al., 2022). However, previous studies have only investigated these relationships separately, overlooking the potential interrelations among teachers' psychological adaptability, work engagement, and digital information literacy self-efficacy. Also, there are scant studies that examine all the key variables together in the field of education. Thus, this is one of the few studies that look at the mediating role of digital information literacy self-efficacy in the relationship between psychological adaptability and work engagement. An SEM approach is also adopted to ensure a rigorous investigation into the topic.

## 2. Literature review and hypotheses

### 2.1. Characteristics of smart teaching

Smart teaching aims at cultivating students' higher order thinking and innovation and creativity abilities that adapt to the times (Nai, 2022). During design and implementation, it is vital to correctly apprehend the orientation of the curriculum objectives and the two major directions of educational model innovation. The curriculum implementation has the characteristics of diversity and selectivity, generativity and developmentality, intelligence and creativity, virtuality and authenticity, and originality. The characteristics of smart teaching are mostly reflected in the following aspects (Xu et al., 2019). First, smart teaching brings about changes in the teaching environment (infinitely rich learning resources, teacher–student interaction anytime, anywhere, and tailored evaluation and feedback) and learning style (deep learning, adaptive learning, personalized learning, distributed learning, and ubiquitous learning). Second, its teaching goals in the era of artificial intelligence emphasize cultivating students' soft skills and core qualities, such as creativity, critical thinking, communication capabilities, cooperation capabilities, time management capabilities, emotional intelligence, self-control, empathy, and caring. Third, in terms of teaching form, smart teaching focuses on the integration and application of new technologies. The first is the rise of micro-classes, MOOCs, and SPOCs, which have reversed traditional teaching, combining “self-learning and teaching,” “online and offline,” and “formal and informal learning.” Next, through the intelligent learning guidance system, students can autonomously follow the notifications to engage in personalized learning activities spontaneously. The emergence of 3D technology, virtual reality (VR), augmented reality (AR), game teaching, and smart classrooms, has optimized and supplemented traditional teaching. Fourth, smart teaching values the process, accuracy, data, and individualization of teaching evaluation.

### 2.2. Psychological adaptability

Psychological adaptability also referred to in the literature as psychological adaptation is defined as “a process of change manifested by individuals in the face of changes in the environment” (Li, 2022, p. 2). According to Martin et al. (2021), adaptability which connotes the capacity to effectively respond to change and numerous work-related tasks is essential for the psychological wellbeing of an individual. Throwing more light on the concept of adaptability and its relation to psychological orientation, Martin et al. (2012) added that adaptability is the ability to “constructively regulate psycho-behavioral functions in response to new, changing, and/or uncertain circumstances, conditions and situations” (p. 66). Thus, adaptability is conceptualized in terms of three psychological dimensions, which include thought, emotion, and action (Martin et al., 2012, 2021). The psychological adaptability of teachers has been considered a significant issue for schools, educators, and students (Collie et al., 2018). This is because psychological adaptability is linked with teacher attrition, stress, and burnout and provides an understanding of how teachers can be supported at work for higher productivity in an era where changing school reforms and students' needs.

Scholars have mainly explored psychological adaptability from the dimensions of school adaptability and evolution of its concepts. Othman et al. (2018) explored positive psychological traits and occupational adaptability, finding that with the advancement of technology, employees need to continuously learn and adapt to the new situation of future work. Career adaptability is a key variable that expresses the ability to self-adjust and adapt to career changes (Othman et al., 2018). The psychological adaptability of teachers is fundamental to their occupational commitment and wellbeing, which ultimately predicts student academic performance (Martin et al., 2012, 2021). Teacher psychological adaptability which includes the feeling that school authorities/leaders are autonomy-supportive has a positive impact on their commitment to their jobs and wellbeing (Holliman et al., 2022).

The above studies all show that psychological adaptability can reveal the effectiveness of an individual's ability to interact well with a changing environment, which is a dynamic process of individual psychological self-regulation. Smart teaching environmental adaptability refers to the teaching adaptability with which teachers overcome various difficulties in the teaching process to obtain excellent teaching skills. Psychological adaptability in the teaching process means that teachers can correctly view the transformation of their educational roles and accurately recognize and evaluate their teaching effects. Good psychological adaptability helps teachers better control the teaching situation during the teaching process, master the teaching process, and achieve the optimal teaching outcome (Waldeck et al., 2021).

### 2.3. Digital information literacy self-efficacy

Digital information literacy refers to the ability to locate, assess, and effectively make use of information to solve problems (Tang and Tseng, 2013). Digital information literacy is an important skill for teachers to cultivate in this intelligent era where there is a call for most educational institutions to infuse smart technologies in schools for instructional delivery. According to Tang and Tseng (2013), there is a correlation between digital information literacy and self-efficacy. Bandura (1986) defined self-efficacy as an individual's sense of belief that he/she can undertake a particular course of action or adequately complete a given task. Human behaviors are primarily impacted by individual capabilities, that is, performance expectations. Hence, self-efficacy is a highly influential factor in accomplishing the task. Guorong and Yusuf (2020) argued that self-efficacy was essentially self-generated capabilities through which people could weigh and judge their skills and evaluate and change their thoughts. Self-efficacy can influence people's attitudes toward difficulties, affect emotions and activities, and influence the acquisition of new behaviors and the performance of acquired behaviors (Guorong and Yusuf, 2020). Bandura (1982) and Bandura et al. (1999) found that teachers' belief in efficacy could influence the direction of the educational process and teaching activities.

Combining both terms, Naveed and Mahmood (2022) defined digital information literacy self-efficacy as “the peoples' beliefs in their ability to successfully recognize when information is needed and have the ability to locate, evaluate, and use effectively the needed information” (p. 1). In this study, we conceptualize the self-efficacy of digital information technology refers to the beliefs and expectations of individuals about their digital information literacy capabilities in the process of achieving their purposes. It, therefore, encompasses teachers' speculations and judgments of their abilities to collect and process their information, as well as judging whether they can successfully finish work tasks in a digital environment during intelligent education and teaching activities.

### 2.4. Work engagement

Schaufeli's (2016) conceptualization of work engagement posits that it is a positive, work-associated, and self-recognizing state of mind, as well as a working state that is the positive opposite of job burnout, characterized by vigor, dedication, and absorption. Vigor means being energetic and adaptable at work, willing to work hard, and persevering even in the face of difficulties. Dedication means vigorously devoting oneself to one's work, experiencing the significance of the work, and feeling enthusiasm and pride (Schaufeli, 2016). Absorption refers to the degree of focus on work; while working, one may feel that time passes quickly and be uneasy about finishing work (Narisada, 2020; Wood et al., 2020).

### 2.5. Relationships among psychological adaptability, digital information self-efficacy, and work engagement

Various studies have analyzed the relationship between teachers' psychological adaptability and work engagement. Dramanu (2020) conceptualized psychological adaptability as psychological flexibility and found it has a significant relationship with work engagement. Psychological adaptability, which also implies a teacher's emotional state and optimism during unfavorable work conditions, was found to correlate with self-efficacy and work engagement (Dong and Xu, 2022). Also, it has been underscored that employees who are adaptive develop self-efficacy and have a high work engagement (Kašpárková et al., 2018). In another study, well-adjusted or resilient teachers (those who are psychologically adaptive) also reported high levels of self-efficacy and work engagement (Perera et al., 2018). Collie et al. (2018) claimed that teacher autonomy was positively correlated with adaptability and negatively correlated with fatigue and disengagement. Moreover, adaptability was negatively correlated with disengagement. Yang et al. (2019) constructed a moderated mediation model and found that work engagement played an intermediary role in psychological adaptation, which might draw people's attention to the possibility of affecting employees' professional adaptability. They also found that when the degree of relationship was low, psychological adaptability had an indirect impact on employee happiness via work engagement. Gupta (2019) built a regression analysis model and found that work engagement could fully mediate the relationship between people's perceived work support and work performance. Yoo and Lee (2019) investigated the mediating role of work insecurity in psychology and work engagement. They found that work insecurity relieved the indirect link between work engagement and self-evaluation through psychological adaptability. According to the previous literature, we propose the following hypothesis:

H1: Under the smart teaching environment, teachers' psychological adaptability is positively related to work engagement.

Research has also studied the relationship between teachers' digital information literacy self-efficacy and work engagement. Teachers who are efficacious in using digital tools for pedagogical practices are often relieved from cognitive burdens, which leads to their higher engagement in work-related activities (Sang et al., 2023). Fute et al. (2022) also cited digital literacy as one of the several factors that affect teacher work engagement. Several studies have also mentioned that during the COVID-19 school closures, the digital literacy of teachers was integral in their work engagement (Gobbi et al., 2021; Yu, 2022). Because of the sudden disruption in education and the impossibility of face-to-face learning, educational delivery was mainly via online platforms. Teachers who were digitally competent were able to use available digital tools for pedagogical instruction. Teachers who are intimidated by technology experience difficulty teaching in technology-enhanced learning environments (Chen, 2017) such as smart educational settings where more advanced technology is implemented for teaching and learning (Kalugina and Tarasevich, 2018; Achilovich, 2021). Digital literacy self-efficacy is crucial to teacher engagement in a digitally rich world (Skantz-Åberg et al., 2022). Teachers with the sense of belief that they have a high command of the use of technology are able to leverage digital tools to change classroom practice both socially and pedagogically. In the study by Deroncele-Acosta et al. (2021), they found a positive relationship between teacher work engagement and digital literacy skills. Drawing on these insights, we propose the following hypothesis:

H2: Digital information literacy self-efficacy is positively related to work engagement.

Regarding research on the relationship between teachers' psychological adaptability and digital information literacy self-efficacy, Qualter et al. (2015) designed different scales to analyze the impact of students' psychological adaptability on self-efficacy. They found that learning with strong psychological adaptability could achieve more achievements in self-efficacy, which was reflected in practice in the improvement of students' adaptability to the environment and their capabilities. To study the correlation between the psychological adaptability and self-efficacy of students under the ever-changing Internet, Keshavarz et al. (2017) developed a more detailed scale and discovered that Iranian graduate students felt high self-efficacy with different dimensions of information literacy. Jiang et al. (2018) discovered that when employees reported long tenure and low work self-efficacy, the correlation between professional adaptability and work content platform was the strongest. Pajic et al. (2018) found that people with higher psychological adaptability were more confident in engaging in job-seeking behaviors in the destination country, and most of them were affected by the enhancement of their self-efficacy. Such a correlation would be weakened when participants encounter higher social barriers but strengthened when participants encounter higher administrative barriers. Atitsogbe et al. (2019) observed that occupational adaptability and general self-efficacy were positively correlated with self-perceived employability. Psychological adaptability made a greater contribution to job seekers, while only general self-efficacy was correlated with entrepreneurial intentions. Drossel et al. (2020) observed that despite their socio-economic challenges, some schools scored better in reading literacy and mathematics because of the positive psychological guidance that colleges and universities afforded students, from which they developed greater self-efficacy in the learning process. Overall, we thus propose the following hypothesis:

H3: Teachers' psychological adaptability is positively related to digital information literacy self-efficacy.

There is significantly less research on the relationships among psychological adaptability, work engagement, and digital information literacy self-efficacy. However, there are a few studies that examined predictors of interrelated concepts of psychological adaptability such as mental or psychological resilience that found technology to mediate the relationship between psychological adaptability and work engagement (Chen et al., 2018; Ojo et al., 2021). Thus, effective technology use means that teachers are efficacious in accessing digital information (i.e., they possess digital information self-efficacy). Ojo et al. (2021) believe that effective technology use in accessing information or for other pedagogical purposes reduces stress which results in psychological adaptability and, in turn, will have an impact on work engagement. Schaufeli et al. (2002, 2008) revealed that employees with higher self-efficacy had high scores on the Utrecht Work Engagement Scale; that is, the employees who had a sense of belief in their competencies had a strong inner drive to rapidly engage in work activities. Salanova and Schaufeli (2008) also found an association between the psychological state (adaptability) of employees and work engagement and a relationship between self-efficacy and work engagement. Keshavarz (2020) also suggested that Internet literacy was also an important skill essential for using Internet resources. They proposed that teachers' work engagement, information-associated skills, and research capabilities depended on their psychological characteristics (Liu et al., 2020). Thus, in this study, we believe that self-efficacy is integral in the link between teacher psychological adaptability and engagement. Therefore, we propose the following hypothesis:

H4: Digital information literacy self-efficacy plays a mediating role in the relationship between psychological adaptability and work engagement.

## 3. Research methods

### 3.1. Research sample

In this cross-sectional research design study, a questionnaire survey was conducted among frontline teachers from six universities in southwest China who were randomly sampled from the target population. The study recruited a sufficiently large sample size given that the preliminary power analysis performed by the G^*^Power Version 3.1.9.2 showed a moderate effect size (*F* = 0.25), with the significance level of α = 0.05, the power was 0.80, and the need of a total sample size of at least 125. The sample in our study was made up of a total of 373 active college professional teachers. All the teachers participated in the survey voluntarily, and they were invited complete a questionnaire. The questionnaire includes a consent form that is approved by the authors' University Survey Research Ethics Committee. The initial sample was made up of 398 teachers from southwestern area of China who were randomly selected from six universities. We identified 25 cases that were removed from the sample for not completing the entire questionnaire or because we found that they had completed it randomly. As the main variable in the study was engagement, the selection of participants included noting their current online teaching working situation. Once the evaluation instruments were selected, and before data collection, the participants in the sample were assured that the study would comply with appropriate standards of data retention, confidentiality, and ethics in how the data would be treated. The questionnaires were applied through a web platform Wenjuanxing, which allowed each subject to complete their part online. In order to check for random or incongruent responses, we included a series of control questions, which would detect those cases and highlight anyone in the sample who responded randomly.

A total of 398 questionnaires were distributed, and 373 valid questionnaires were collected, for an effective response rate of 94%. We analyzed the sample characteristics of teachers and used SPSS software to organize the data characteristics. In terms of gender distribution, 144 (38.6%) participants are male and 229 (61.4%) female. In terms of disciplines, 56 participants (15%) are art teachers, 132 (35.4%) are management teachers, and 185 (49.6%) are science teachers. In terms of job titles, 103 (27.6%) have junior titles, 223 (59.8%) have intermediate titles, and 47 (12.6%) have senior titles. In terms of teaching experience, among the 372 participants, 103 (27.6%) have 0–5 years of teaching experience, 135 (36.2%) have 6–10 years, 85 (22.8%) have 11–15 years, and 50 (13.4%) have over 16 years of teaching experience.

### 3.2. Survey methods

The Work Engagement Scale (WES), the Teacher Information Technology Teaching Psychological Adaptability Scale (TITTPAS), and the Teacher Digital Information Literacy Self-Efficacy Scale (TDILSES) were used for investigation and analysis. Here, teachers' psychological adaptability refers to college teachers' psychological and behavioral responses in the smart teaching and online teaching environment to their teaching, scientific research, support environment, and physical and mental changes, which will be divided into four dimensions: adaptability of ideas and attitudes, adaptability of capabilities, adaptability of support environment, and adaptability of behaviors. The Work Engagement Scale (WES; Loscalzo and Giannini, 2019), the Teacher Information Technology Teaching Psychological Adaptability Scale (TITTPAS; Collie et al., 2020a,b), and the Teacher Digital Information Literacy Self-Efficacy Scale (TDILSES; Yavuzalp and Bahçivan, 2020) include 45 questions in these four dimensions.

The TDILSES is adapted from the Information Literacy Self-Efficacy Scale, the Teacher Information Literacy Self-Efficacy Questionnaire, and the higher education information literacy standard promulgated by the National Library of America. Based on the localized translation, 28 high-reliability questions along four dimensions were compiled for teachers under the smart teaching environment.

Usluel (2007) researched teacher information literacy self-efficacy, finding that the level of using ICT and the length of use time were the determinants of self-efficacy. It, therefore, seems that the teaching and learning environment in the intelligent education era makes teachers improve their information literacy and cultivate high information literacy self-efficacy. Information literacy self-efficacy determines how much effort teachers have to put into their development, how long they will persist, and their attitude when facing difficulties. The 28 questions are scored on a 5-point Likert scale. The internal consistency of this instrument is a Cronbach's alpha value of 0.82.

The WES uses the full version of the Utrecht Work Engagement Scale (UWES). This scale includes three dimensions: vigor, dedication, and absorption, with a total of 17 questions, all scored on a 7-point Likert scale. The vigor sub-scale is made up of six items (e.g., “When studying I feel strong and vigorous.”). Five items comprise the dedication subscale (e.g., “I find my studies to be full of meaning and purpose.”). And, the absorption subscale is made up of six (e.g., “I get carried away when I am studying.”). The internal consistency of the UWES-17 is good with Cronbach's alpha value equal to or exceeding the critical value of 0.70. Specifically on the subdimensions of the UWES-17, vigor is characterized by high energy at work, strong mental flexibility, willingness to work hard, and perseverance even when encountering difficulties. The characteristic of dedication is full devotion to work and the experience of a sense of meaning, enthusiasm, pride, and challenge. The characteristic of absorption is the ability to devote oneself wholeheartedly to one's work. Although time flies quickly, it is difficult for concentrated people to get rid of work. As this scale is a representative measure of work engagement worldwide, it is used in this study.

### 3.3. Data analysis

SPSS 25.0 and Analysis of Moment Structure (AMOS) 25.0 software were chosen for data analysis. Descriptive statistics and correlation analyses were conducted using SPSS software, while the confirmatory factor analysis, structural equation model, and mediating effect tests were conducted using AMOS software (Schreiber et al., 2006). The confirmatory factor analysis tests the structural validity of the scale, and the structural equation model tests the hypotheses. The confirmatory factor analysis and structural equation model analysis use the following indicators of the goodness of fit: the chi-square statistic (χ^2^) and a related measure (χ^2^/*df* ), the root mean square error of approximation (RMSEA), the Tucker–Lewis Index (TLI), and the comparative fit index (CFI). The standard threshold criteria of χ^2^/*df* < 3, RMSEA < 0.05, TLI > 0.9, and CFI > 0.9 were adopted to evaluate model fit. Mediating effects were tested by the bootstrap method.

### 3.4. Reliability and validity tests of the scale

The reliability and validity of the three scales were tested. Cronbach's α coefficients of the four dimensions corresponding to the TITTPAS are within the range of 0.88–0.92. Cronbach's α coefficient of TDILSES is 0.96, while the coefficients for the three dimensions of the WES range from 0.86 to 0.91. As Cronbach's α coefficients of all the above dimensions are >0.7, the reliability of the three scales is good. The validity of the three scales was tested by confirmatory factor analysis (CFI), yielding the fit indices for the model of χ^2^/*df* = 1.764, RMSEA = 0.043, CFI = 0.965, TLI = 0.961, showing a good model fit.

## 4. Research results

### 4.1. Descriptive statistics and correlation analysis

As shown in Table 1, the average scores of the four dimensions (ideas and attitudes, capabilities, support environment, and behaviors) of the TITTPAS are >3.9, indicating that the surveyed teachers' psychological adaptability scores are high. Among them, the behavior dimension has the highest average score (*M* = 4.32, *SD* = 0.50). The average TDILSES score is 3.85 (*SD* = 0.59). However, the scores for all three dimensions of WES (vigor, dedication, and absorption) are lower, with the average score for dedication the highest (*M* = 3.63, *SD* = 1.16), followed by vigor (*M* = 2.63, *SD* = 1.10) and absorption (*M* = 2.26, *SD* = 0.89).

**Table 1:** Results of descriptive statistics and correlation analysis.

	**1**	**2**	**3**	**4**	**5**	**6**	**7**	**8**
Ideas and attitudes	–							
Capabilities	0.141^**^	–						
Support environment	0.096	0.003	–					
Behaviors	0.066	0.028^*^	0.048	–				
Self-efficacy	0.222^**^	0.142^**^	0.281^**^	0.155^**^	–			
Vigor	0.202^**^	0.173^**^	0.180^**^	0.283^**^	0.294^**^	–		
Dedication	0.218^**^	0.055	0.01	0.035	0.250^**^	0.065	–	
Absorption	0.175^**^	−0.033	0.246^**^	0.015^*^	0.265^**^	0.036	0.166^**^	–
Mean value	3.95	3.94	4.25	4.32	3.85	2.63	3.63	2.26
Standard deviation	0.57	0.57	0.54	0.50	0.59	1.10	1.16	0.89
Cronbach's alpha	0.89	0.92	0.89	0.88	0.96	0.89	0.86	0.91

* p < 0.05.

** p < 0.01.

Table 1 also presents the correlation matrix of the eight dimensions and their significance. The four dimensions of the TITTPAS have a significantly positive correlation with self-efficacy, and the ideas and attitudes dimension of the TITTPAS has significantly positive correlations with the three dimensions of the WES. The capabilities dimension of the TITTPAS only has a significantly positive correlation with the vigor dimension of WES. The support environment dimension of the TITTPAS has significantly positive correlations with the dimensions of vigor and absorption of the WES. Finally, the behaviors dimension of the TITTPAS only has a significantly positive correlation with the vigor dimension of the WES. The above results reveal a significant positive correlation between self-efficacy and the three dimensions of the WES.

### 4.2. Structural equation modeling results

**Figure 1:**
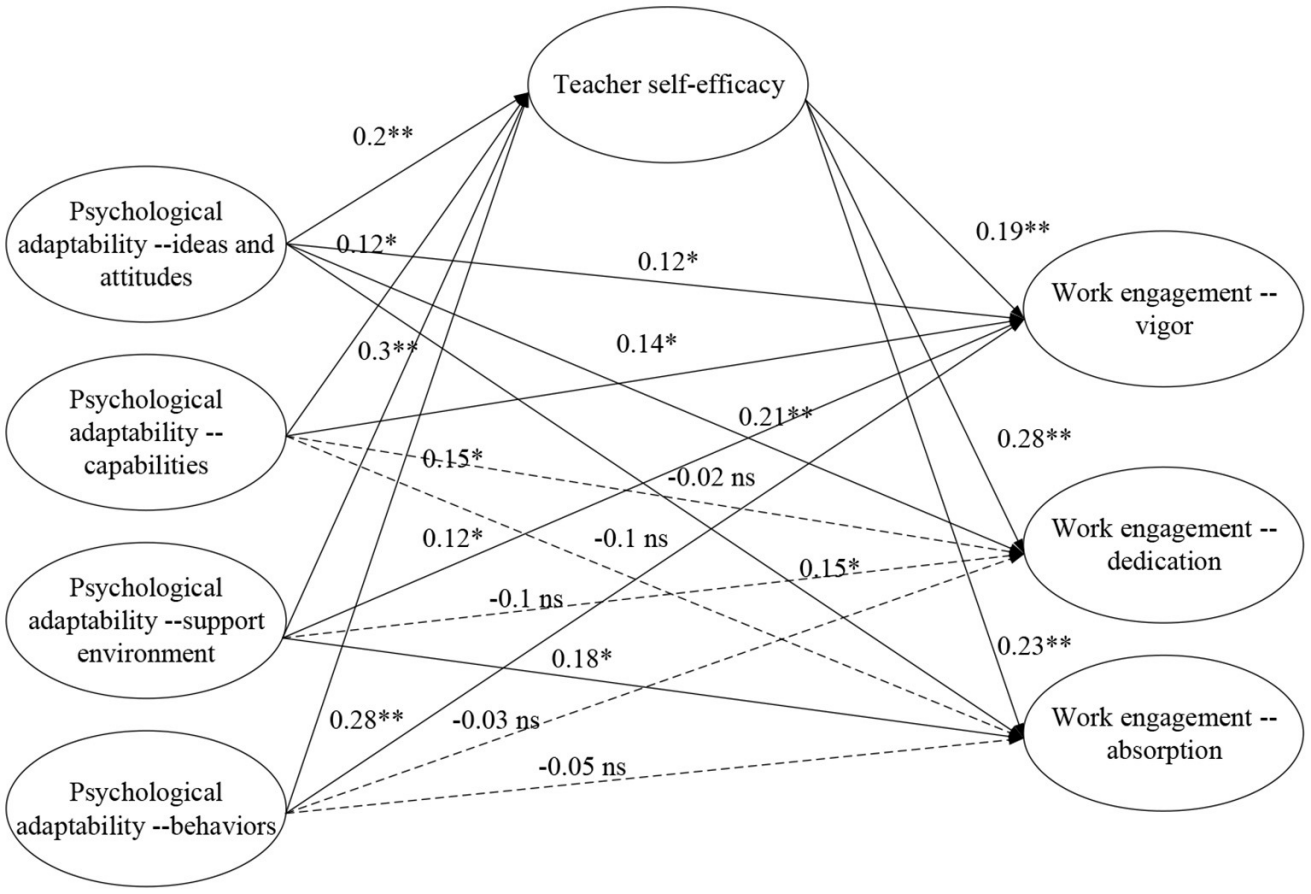
Structural equation modeling results.

The hypotheses were tested using the structural equation model, and the results are presented in Figure 1. The model shows a good fit to the data (χ^2^/*df* = 1.785, RMSEA = 0.046, CFI = 0.934, and TLI = 0.926). The model analysis results show that teachers' ideas and attitudes have a significant positive effect on self-efficacy (β_1_ = 0.19, *p*_1_ < 0.01); their capabilities have a significant positive effect on self-efficacy (β_2_ = 0.12, *p*_2_ < 0.05); the support environment has a significant positive effect on self-efficacy (β_3_ = 0.20, *p*_3_ < 0.01); and behavior has a significant positive effect on self-efficacy (β_4_ = 0.15, *p*_4_ < 0.05). Among the dimensions of teacher self-efficacy, vigor has a significant positive impact on work engagement (β_1_ = 0.19, *p*_1_ < 0.01); dedication has a significant positive impact on work engagement (β_2_ = 0.28, *p*_2_ < 0.05); and absorption has a significant positive impact on work engagement (β_3_ = 0.23, *p*_3_ < 0.01). The ideas and attitudes in teachers' psychological adaptability have a significant positive impact on vigor in work engagement (β_1_ = 0.12, *p*_1_ < 0.05). Dedication in work engagement has a significant positive effect (β_2_ = 0.15, *p*_2_ < 0.05), and ideas and attitudes have a significant positive effect on absorption in work engagement (β_3_ = 0.21, *p*_3_ < 0.01). Teacher capabilities only have a significant positive effect on vigor in job engagement (β = 0.14, *p* < 0.05). The support environment has a significant positive effect on vigor in work engagement (β_1_ = 0.12, *p*_1_ < 0.05) and a significant positive effect on absorption in work engagement (β_2_ = 0.18, *p*_2_ < 0.01). Teacher behavior only has a significant positive effect on vigor in work engagement (β = 0.28, *p* < 0.01).

### 4.3. Bootstrap test of mediating effects

Among the four mediating paths of the four dimensions of teacher psychological adaptability through the mediating variable self-efficacy to the vigor dimension of work engagement, the paths of ideas and attitudes, support environment, and behaviors are all significant; in contrast, the mediating path of capabilities through self-efficacy is not significant. Moreover, because ideas and attitudes, support environment, and behaviors have significant and direct effects on vigor, these three dimensions have partial mediating effects. All four of the mediating paths of dedication are significant, and among them, ideas and attitudes have a significant and direct effect on dedication. Therefore, the mediating effect of ideas and attitudes on dedication through self-efficacy is partial (Table 2). Contrariwise, the direct effects of capabilities, support environment, and behaviors on dedication are not significant; thus, they have full mediating effects on dedication through self-efficacy. Among the four mediating paths involving absorption, the mediating effect of capabilities through self-efficacy is not significant, and capabilities have no significant direct effect on absorption. Ideas and attitudes and support environment have significant direct effects and significant mediating effects on absorption; therefore, these two mediating effects are partial. Behaviors have no significant direct effect on absorption; however, the mediating effect is significant. Therefore, behaviors have a full mediating effect on absorption through self-efficacy.

**Table 2:** Bootstrap test results of mediating effect.

**Dependent variable**	**Independent variable**	**Direct effect**	**Mediating effect analysis**			
			**Mediating effect**	**95% Bootstrap CI**		***p*** **-value**
				**Lower**	**Upper**	
Vigor	Ideas and attitudes	0.122^*^	0.037	0.006	0.078	0.017
	Capabilities	0.135^*^	0.023	−0.001	0.064	0.055
	Support environment	0.117^*^	0.056	0.013	0.101	0.007
Behaviors	0.276^**^	0.028	0.002	0.071	0.022
Dedication	Ideas and attitudes	0.205^*^	0.057	0.012	0.118	0.011
	Capabilities	−0.022	0.035	0.001	0.08	0.049
	Support environment	−0.103	0.085	0.035	0.145	0.001
Behaviors	−0.025	0.043	0.005	0.099	0.017
Absorption	Ideas and attitudes	0.149^*^	0.046	0.007	0.103	0.014
	Capabilities	−0.098	0.028	−0.001	0.065	0.052
	Support environment	0.181^**^	0.069	0.023	0.122	0.005
Behaviors	−0.051	0.035	0.003	0.083	0.020

* p < 0.05.

** p < 0.01.

## 5. Discussion

In this study, we aimed to analyze the relationships among college teachers' psychological adaptability, work engagement, and digital information self-efficacy. To do so, we draw on a sample of frontline teachers and conduct a structural equation model analysis.

The results of structural equation modeling demonstrate that perception and attitude of the dimension of psychological adaptability had a significant positive impact on each dimension of work engagement. The results of this study are consistent with Hypothesis H1 regarding the influences of teachers' psychological adaptability, job engagement, self-efficacy, and digital information relationship, showing that teachers' psychological adaptability is positively correlated with job engagement, corroborating a previous study (Xiong et al., 2020). Job satisfaction has a certain impact on employees' work enthusiasm, professional cognition, professional commitment, and job burnout and even has a certain impact on their life outside work. Psychological adaptability affects teachers' internal satisfaction. In addition, psychological adaptability partially mediates the relationship between psychological capital and teachers' internal job satisfaction. In this study, psychological adaptability has a positive predictive effect on job satisfaction. Meanwhile, positive psychology has a direct primary predictive effect on job satisfaction and indirectly affects job satisfaction through psychological adaptability as an intermediary. Psychological adaptation partially mediated the relationship between positive psychology and job satisfaction. On the one hand, positive psychology affects the level of psychological adaptability. On the other hand, the level of psychological adaptability affects the level of job satisfaction. Tjin A Tsoi et al. (2018) studied psychological needs and motivational adaptations and developed a structural equation model to analyze the relationship between pharmacists' basic psychological needs, wellbeing, and learning outcomes. The findings suggested that factors such as training status and work experience influenced the observed learning motivation scores, showing that organizational leadership support, a good software and hardware environment, and harmonious interpersonal relationships had a significant stimulating effect on teachers' work vitality and concentration. However, teaching ability and behavior only had a significant positive effect on vitality but not on dedication and focus. This is consistent with our findings.

Teachers, like all individuals, are driven by both intrinsic motivation and extrinsic motivation, and they experience a high-energy state in their work and have a high degree of identification with their work, resulting in strong work motivation. Therefore, they experience a high sense of work engagement. We can deeply analyze which reasons drive teachers to have a high level of work engagement based on the reasons why teachers work hard. Regarding the relationship between teachers' digital information literacy self-efficacy and job engagement, this study found that teachers' digital information literacy self-efficacy was significantly positively correlated with job engagement. Teachers' digital information literacy self-efficacy reflects their evaluation and judgment of their ability to complete digital information teaching and intelligent teaching tasks. This finding is consistent with Hypothesis H2 (Digital information literacy self-efficacy is positively related to work engagement) and is consistent with previous studies (Tang and Tseng, 2013; Kultawanich et al., 2015; Naveed and Mahmood, 2022; Shonfeld et al., 2022). For example, Shonfeld et al. (2022) found that teacher information literacy self-efficacy was found to correlate with their digital skills and their participation in digital programs. Thus, teachers have to develop their digital skills to be efficacious in guiding students in smart learning environments where students interact with digital tools in the teaching and learning process. The self-efficacy and digital information literacy of learners (in our context teachers) can affect their self-confidence and teaching performance (Tang and Tseng, 2013). While technology offers good learning opportunities in smart learning environments, the inability to effectively use it for pedagogical purposes can stress teachers and affect student learning outcomes. It is therefore essential for teachers to develop their digital literacy skills to build confidence in their ability to facilitate learning. Teachers will be more proactive to engage in digital teaching if they have a sense of belief that they have an adequate command of technology. Sang et al. (2023) observed that the digital competence of teachers had a positive correlation with their work engagement.

The higher their self-efficacy, the more confident teachers are in their own abilities. In the task of online teaching, teachers tend to take proactive actions and devote themselves to teaching. Positive actions and efforts will produce positive work input results, stimulate teachers' work vitality, improve teachers' work engagement, and promote teachers' work absorption. In a complex intelligent teaching environment, teachers with low self-efficacy present relatively negative attitudes and behaviors, which will directly affect their work engagement, leading to unsatisfactory job performance and even job burnout. As a result, their interest in and recognition of teaching work will suffer, reducing work engagement. At the teacher's level, teachers who use information technology in teaching must accept information-based teaching in terms of concepts and attitudes and have the relevant ability to adapt to information-based teaching. They also need to provide corresponding support environments for teachers' information-based teaching, such as hardware, software, and interpersonal relationships. The change in teachers' teaching behavior using information technology is primarily determined by the various abilities required for the use of information technology in teaching, followed by concept adaptation, and attitude adaptation is the least decisive. The adaptation of the teaching support environment has no obvious decisive effect on changes in teachers' teaching behavior using information technology. However, the teaching support environment is a necessary condition for teachers to implement and carry out information-based teaching. Thus, it should also receive attention from schools and education-related departments.

The findings on the relationship between teachers' psychological adaptation and digital information literacy self-efficacy found that teachers' psychological adaptation (concepts and attitudes, abilities, supportive environments, and behaviors) had a significant positive impact on self-efficacy. Two dimensions of psychological adaptation were significantly positively correlated with self-efficacy: attitudes and supportive environments. The results of this study partially confirmed Hypothesis H3 (Teachers' psychological adaptability is positively correlated with digital information literacy self-efficacy). Moreover, Usluel's (2007) study of the self-efficacy of teachers' information literacy found that the teaching and learning environment in the era of intelligent education enables teachers to improve their information literacy and cultivate the self-efficacy of college teachers' digital information literacy. It can be found that the psychological adaptability of teachers in the era of intelligent education is positively correlated with the self-efficacy of digital information, which is similar to the findings of this study on the relationship between teachers' psychological adaptability and digital information literacy self-efficacy that confirm the positive relationship between teachers' digital information literacy self-efficacy and psychological adaptability. Overall, there are significant positive correlations between psychological adaptation and general self-efficacy to varying degrees, which is basically consistent with Bingöl et al.'s (2018) research results. In their study, psychological adaptation was conceptualized as psychological resilience. They discovered that psychological resilience and general self-efficacy have a positive adaption. The improvement of general self-efficacy is conducive to improving the level of psychological adaptation of college teachers. This verifies the hypothesis that it would show a positive correlation with the psychological adaptation of college teachers.

Psychological adaptation refers to the process whereby the subject makes an active response through the self-regulation system when the external environment changes. When the subject and the environment reach a new balance, psychological adaptation is a process of change manifested by the individual in the face of changes in the environment. The bootstrapping test of the mediation effect explored the relationship between psychological adaptability, job engagement, and digital information literacy self-efficacy, showing that the four dimensions of college teachers' mental health have different effects on different aspects of job engagement and digital information literacy self-efficacy. The results of this study thus confirmed Hypothesis H4. Holliman et al. (2022) developed and tested a conceptual model of a predictive relationship between university lecturer fitness and mental health, using structural equation modeling to model the link. These studies are consistent with our findings, suggesting that although adaptation is important and that its impact may be more pronounced in higher education. The findings extend previous research on school teachers, showing that, while adaptability is important, its impact may be pronounced in pre-tertiary education, where higher regulation and lower autonomy are often present. Teachers with high levels of self-efficacy are more confident in their own work ability, feel more positive emotions, dare to face difficulties and accept challenges, and are less affected and troubled by negative emotions. This state helps teachers maintain a good physical and mental condition, adapt to the working environment, coordinate interpersonal relationships, resolve negative emotions generated at work, and maintain a positive working state. In addition, teaching and research staff with low self-efficacy will underestimate their own abilities and see tasks as more difficult and obstacles as greater than they actually are, resulting in increased work pressure and even work burnout. As a result, it is difficult for them to maintain positive work engagement.

The results of the bootstrapping test of the mediation effects suggest that teachers' psychological adaptability (ideological attitude and support environment) has a significant direct impact on job engagement. Meanwhile, teachers' psychological adaptability (ideological attitude and support environment) and job engagement have a significant direct impact on digital information literacy self-efficacy (behavior and ability). Therefore, teachers' psychological adaptability (ideological attitude and support environment) has an indirect influence on digital information literacy self-efficacy (ability). When studying the relationship between digital information literacy self-efficacy (behavior and competence), job engagement (vigor, dedication, and absorption), and teachers' psychological fitness (ideological attitude and supportive environment), it was found that teachers' ideology and attitudes are related to dedication. Therefore, the effect of teachers' psychological adaptability on job engagement through teachers' digital information literacy self-efficacy is indirect. However, the direct influence of ability, support environment, and behavior on dedication is not significant. Thus, digital information literacy self-efficacy plays a mediating role in the relationship between psychological adaptability and job engagement, but the direct influence of the role is not significant. The direct effect of ability on absorption is not significant, while ideological attitude and support environment have a significant direct effect and significant mediating effect on absorption. Therefore, the mediating effect of digital information literacy self-efficacy on psychological adaptability and job engagement is localized. To sum up, digital information literacy self-efficacy plays a mediating role in the relationship between psychological adaptability and job engagement, but this mediating role is partial and one-sided.

## 6. Implications and conclusion

Our findings also hold important implications for policymakers. First, our findings highlight the importance of providing training for teachers on information technology teaching and of implementing measures to enhance teachers' digital information literacy self-efficacy. Training should focus on the knowledge and skills of teachers using information technology teaching and increase the practical links of teachers using information technology teaching. Moreover, during training, the differences in “idea,” “attitude,” “ability,” “teaching support environment,” and “behavior change” between teachers of different backgrounds should be considered while using information technology. The universities should create a favorable environment to strengthen blended teaching and smart teaching platform training and offer lectures about information technology. In doing so, and in order to achieve a more effective and student-centered environment, cross-departmental cooperation needs to be fostered in building digital informatized skills training platforms, organizational support capabilities should be strengthened, and teachers should be encouraged to use information technology to teach innovation and provided with appropriate support and incentive measures.

In addition, it would be convenient to conduct systematic mental health education to improve teachers' psychological adaptability in complicated digital information teaching and learning environments. Unlike traditional classroom teaching with a fixed model, smart education methods, such as online teaching, MOOC, micro-teaching, and blended teaching, pose new challenges for teachers. The systematic mental health education courses and training should be designed in a targeted manner, while individual mental health counseling should be focused on as well. Flexible and diverse forms of mental health services should be provided to college teachers to improve their psychological adaptability, thereby helping them better face and solve the problems encountered in individual development.

Furthermore, both teacher work engagement and their psychological adaptability affect the academic achievement of students (Han and Wang, 2021). Thus, the resilience of teachers during adverse situations and their ability to happily concentrate on their work shape how students learn. Teachers are the forerunners of education and the immediate contact point for students. In this light, educators and policymakers in education seeking to promote good learning outcomes for students should endeavor to ensure teachers are actively engaged in their work and are in the right mental state to instruct students. From a theoretical implications point of view, researchers should actively investigate factors that affect teacher work engagement and their psychological adaptability. Practically, guidance and counseling units can be constructed in schools that monitor the psychological dispositions of teachers and ensure their emotional health is catered for. Also, issues relating to remuneration, good working conditions, supply of good teaching materials, etc., should be appropriately addressed for teachers to develop mental fortitude and happiness when instructing students.

We acknowledge that our study is subject to some limitations. First, our findings are based on a sample from a single country, and therefore, generalizations to other similar contexts must be done with caution. Moreover, this study did not evaluate teachers' mental health with regard to teachers' psychological adaptability in a complex digital information teaching environment. It is essential to perform a reasonable analysis, including bootstrap tests of the mediating effect for the modeling of structural equations. Second, the data were gathered by self-reporting and personal experience, which may mean contamination by the common method variance. It would be useful to complement these results with other measures gathered by other methods. Also, there are potential demographic variables such as age which might have an extraneous effect on digital literacy self-efficacy and psychological adaptability, but they were unexplored in this study. Future researchers can examine the effect of demographic variables on the study variables. Another limitation is that all measures were self-reported and may be suffered from common method bias. Third, the study performed a cross-sectional research design that does not provide a causal relationship among the variables. Overall, and despite the aforementioned limitations, we believe that our study contributes to the growing body of literature focused on smart teaching environments. However, we also stress that more efforts are needed to enlarge our understanding of this important phenomenon, both theoretically and empirically. We hope our research will encourage other scholars to pursue this fascinating line of investigation.

## Data availability statement

The original contributions presented in the study are included in the article/supplementary material, further inquiries can be directed to the corresponding author.

## Ethics statement

The studies involving human participants were reviewed and approved by Southwest University Survey Research Ethics Committee. The patients/participants provided their written informed consent to participate in this study.

## Author contributions

SF: design the research, collect the data, and write the draft. XZ: design the research, analyze the data, and revise the draft. ZY: conceptualize the framework and collect data. CG: revised the manuscript and approve the final version of the manuscript. All authors contributed to the article and approved the submitted version.
